# Liver Fibrosis and Hepatitis B Coinfection among ART Naïve HIV-Infected Patients at a Tertiary Level Hospital in Northwestern Tanzania: A Cross-Sectional Study

**DOI:** 10.1155/2017/5629130

**Published:** 2017-07-30

**Authors:** Semvua B. Kilonzo, Daniel W. Gunda, Flora Kashasha, Bonaventura C. Mpondo

**Affiliations:** ^1^Department of Medicine, Catholic University of Health and Allied Sciences, P.O. Box 1464, Mwanza, Tanzania; ^2^Department of Medicine, Bugando Medical Centre, P.O. Box 1370, Mwanza, Tanzania; ^3^Department of Medicine, College of Health Sciences, The University of Dodoma, P.O. Box 395, Dodoma, Tanzania

## Abstract

**Background:**

Liver fibrosis which is a common complication of chronic hepatitis B infection is rarely diagnosed in low-resource countries due to limited capacity to perform biopsy studies. Data on the utilization of noninvasive techniques which are feasible for diagnosis of liver fibrosis in these settings among HIV-infected patients is scarce. The objective of this study was to establish the magnitude of liver fibrosis by using both aspartate-aminotransferase-to-platelets ratio and fibrosis-4 scores with associated hepatitis B coinfection among antiretroviral therapy naïve HIV-infected patients.

**Methods:**

We reviewed data of 743 adult patients attending HIV clinic with available hepatitis B surface antigen test results. Baseline clinical information was recorded and aspartate-aminotransferase-to-platelet ratio and fibrosis-4 scores were calculated. The cut-off values of 1.5 and 3.25 were used for diagnosis of significant fibrosis by aspartate-aminotransferase-to-platelets ratio and fibrosis-4 scores, respectively.

**Results:**

The prevalence of liver fibrosis was 3.5% when aspartate-aminotransferase-to-platelet score was used and 4.6% with fibrosis-4 score and they were both significantly higher among patients with hepatitis B coinfection. Younger patients with HIV advanced disease and elevated liver transaminases had increased risk of having hepatitis B coinfection.

**Conclusion:**

A remarkable number of HIV-infected patients present with liver fibrosis, predominantly those with hepatitis B infection.

## 1. Introduction

Hepatitis B virus (HBV) and human immunodeficiency virus (HIV) coinfections are common. Up to 10% of 33 million people living with HIV worldwide have been affected by HBV infection [1]. According to a recent meta-analysis survey, the rate of coinfection is ranging from 0% to as high as 28% in sub-Saharan Africa (SSA) with higher rates being reported in West African countries (median: 11.5%); East African countries were found to have the lowest rates (median: 4.1%) [2]. The prevalence of HBV/HIV coinfection in Tanzania is 6.2% [3].

A profound impact in both hepatitis and HIV diseases' progression has been attributed to the coinfections. Rapid deterioration to liver cirrhosis and hepatocellular carcinoma (HCC) with an increased mortality is a major consequence of HIV infection in chronic hepatitis B [4]. Likewise, HBV infection has been correlated with several clinical manifestations in HIV-infected patients including impaired immune response during antiretroviral therapy (ART) and increased susceptibility to ART-related liver toxicity [5]. These interactions uphold the importance of timely screening of one infection in the presence of the other, with a consequent detection of associated complications at their early stages.

The diagnosis of liver fibrosis/cirrhosis is challenging and usually delayed in resource-limited settings due to unavailability of invasive biopsy studies that is being considered as a gold standard test. Liver transaminases which are the commonly used markers in these settings are less efficient for diagnosis of liver disease in HIV-infected patients [6]. World Health Organization (WHO) has recently recommended the use of noninvasive tests (NITs): aspartate amino transferase- (AST-) to platelets ratio index (APRI) and fibrosis-4 (FIB-4) scores for estimation of significant liver fibrosis in resource-limited settings [7]. These scores require the routinely done hematological and serological laboratory tests like AST, alanine amino transferase (ALT), and platelets. Data on utilization of APRI and FIB-4 scores for detection of liver fibrosis in HIV-infected individuals in SSA is scarce. There is also limited data on magnitude and patterns of liver fibrosis/cirrhosis which is a major complication of chronic hepatitis B (CHB) infection among HIV-infected patients in sub-Saharan Africa (SSA). Few studies have been done in East Africa; studies from Uganda and Morogoro, Tanzania, have documented the prevalence rates of 17% and 9.1%, respectively [8, 9].

In this cross-sectional study, we reviewed data of ART naïve HIV-infected patients registered at HIV care and treatment clinic, in the northwestern zonal referral hospital in Tanzania. The objective of this study was therefore to establish the magnitude of liver fibrosis by using APRI and FIB-4 scores and to assess the rate of associated HIV and HBV coinfection among HIV-infected patients.

## 2. Materials and Methods

### 2.1. Study Design

This was a clinic-based cross-sectional study done at Bugando Medical Centre (BMC) HIV care and treatment clinic (CTC) between 1 May 2014 and 31 July 2015.

### 2.2. Study Setting

The study was conducted at Bugando CTC in Mwanza, Tanzania. BMC is a tertiary and teaching hospital for the Lake Zone of Tanzania which serves around 13 million people. The hospital runs both inpatient and outpatient treatment activities, with an approximate bed capacity of 900. CTC activities which are one of the core parts of outpatient activities started in 2004, and they currently serve more than ten thousand patients, of whom about 1000 are ART naïve.

### 2.3. Participants and Samples

We retrospectively reviewed the baseline clinical records of all ART naïve patients who were attending the CTC clinic from 01 May 2014 to 31 July 2015. Patients were excluded if they were younger than 18 years and if hepatitis B antigen surface (HBsAg) test was missing. Patients' age, gender, and marital status were reviewed. Also, the baseline clinical information like HIV stage, CD4 count, HBsAg, ALT, AST, and platelets levels was also recorded on structured questionnaires. APRI and FIB-4 scores were calculated by using standard formulae [10]. Both of these scores have high and low cut-off points for maximizing the accuracy of diagnosis. In APRI, a high cut-off of 2.0 is used to identify patients with cirrhosis which is equivalent to Metavir liver fibrosis score of 4, while a low cut-off of 1.0 rules out the cirrhosis. A significant fibrosis (Metavir ≥ 2) in APRI score is predicted by a cut-off of 1.5 and is ruled out if the score is <0.5. For the case of FIB-4 score, a cut-off point of 3.25 is used to predict significant fibrosis (Metavir ≥ 2) and it is ruled out if the FIB-4 score is <1.45 [7]. In the index study, only high cut-offs were used for each of the tests for significant fibrosis and for APRI to identify cirrhosis. The upper limit of AST was 40 IU/l while that of ALT was 41 IU/l and all values above these cut-off points were coded as elevated liver enzymes.

### 2.4. Statistical Analysis

Data was entered, verified, and cleaned using Microsoft Excel and analysis was done using STATA version 14 (College Station, Texas). Categorical variables were described as proportions, while continuous variables were described as medians and interquartile ranges. Differences in baseline characteristics between patients with and without HBV infection were assessed using Chisquare or Fisher's exact tests, as indicated, for categorical data and Wilcoxon ranksum tests for continuous data. The comparison of APRI and FIB-4 scores among HIV monoinfected and HIV/HBV coinfected patients was presented as unadjusted odds ratios.

### 2.5. Ethical Statement

The permission to conduct this study was obtained from the administrations of Bugando Medical Centre and Catholic University of Allied and Health Sciences (CUHAS) and the ethical clearance was obtained from CUHAS/BMC joint Committee of Research and Publications. No patients' identifiers were used to maintain confidentiality. All the data was handled by researchers.

## 3. Results

### 3.1. Study Enrollment

This study was conducted at Bugando Medical Centre from 01 May 2014 to 31 July 2015. During this period, 4789 adult patients were seen at CTC. Of these, 1210/4789 (25.3%) were ART naïve. Of the ART naïve patients, 743/1210 (61.4%) were enrolled in the study: 467 were missing their HBsAg results.

### 3.2. Baseline Characteristics

From 1 May 2014 to 31 July 2015, a total of 743 HIV-infected ART naïve patients were enrolled. Among those, females were 484 (65.1%) and the median age was 37 (IQR 30–44) years. Majority (386) (51.9%) of these patients had less severe HIV disease (WHO stage 1 or 2) with the median baseline CD4 count levels of 256 (IQR 123–458) cells/*μ*l. The median values of aspartate amino transferase and alanine amino transferase were 26 (IQR 19.5–37) IU/l and 19 (IQR 13–30) IU/l, respectively (Table 1).

### 3.3. HBV Coinfection

Out of 743 patients, 49 (6.6%) were coinfected with Hepatitis B virus with a male: female ratio of 1 : 1.9. These patients were significantly younger (<39 years) (59.2% versus 55.5%,  *P* = 0.002) with lower baseline CD4 cells level of <200 cells/*μ* (67.4% versus 31.4%, *P* < 0.001) as compared to HIV monoinfected group. Liver enzymes, both AST and ALT, were significantly elevated from their cut-off values of 40 IU/l and 41 IU/l, respectively, in HIV/HBV coinfected group (48.5% versus 33.1%, *P* = 0.03, for AST and 42.9% versus 26.2%, *P* = 0.01, for ALT). Majority (61.2%) of the HBV coinfected patients had advanced HIV disease (WHO stage 3 or 4) as compared to their HIV monoinfected counterparts with the odds ratio of 1.77 (0.98–3.21), *P* = 0.006 (Table 1).

### 3.4. Prevalence of Liver Fibrosis by APRI and FIB-4 Scores

A total of 456/743 (61.4%) patients had their laboratory results available for assessment of liver disease. Eleven patients out of 456 (2.4%) had significant liver fibrosis by both APRI and FIB-4 scores, while both tests agreed in 430 (97.7%) of the negative results (APRI < 1.5 and FIB-4 < 3.25). Of the remaining 15/456 (3.3%) patients with discordant APRI and FIB-4 results, 5 (31.3%) were APRI-positive but FIB-4 negative, while 11 (66.7%) were APRI-positive but FIB-4 negative. Twenty-six patients (5.7%) had at least one positive criterion for liver fibrosis. Thus, the overall prevalence of liver fibrosis was 16/456 (3.5%) by using APRI score and 21/456 (4.6%) by FIB-4 score. The agreement between APRI and FIB-4 results was excellent (Kappa = 0.967) (Table 2). When APRI score was used, the prevalence of liver fibrosis among HIV monoinfected patients was found to be 11 (2.58%) while that of HBV/HIV coinfected patients was 5 (17.24%) [OR: 7.88 (2.53–24.49), *P* < 0.001]. The median APRI score was also found to be significantly higher in HBV/HIV coinfected patients as compared to HIV monoinfected group [0.45 versus 0.28, OR: 2.21 (1.44–3.38), *P* < 0.001]. When FIB-4 score was used, patients with HBV/HIV coinfection were also more likely to have liver fibrosis than their HIV monoinfected counterparts (OR = 1.59, 95% CI = 0.35–7.18, *P* = 0.55). The median FIB-4 score values were also higher in coinfected group [1.34 versus 0.96, OR: 1.24 (1.01–1.51), *P* = 0.04]. A total of 10/456 (2.2%) patients were found to have liver cirrhosis (APRI > 2.0) (Table 3).

## 4. Discussion

This cross-sectional study was conducted to establish the magnitude of liver fibrosis and associated HBV coinfection among HIV-infected patients. We have reported a prevalence of coinfection to be 6.6% and that of liver fibrosis by APRI and FIB-4 scores was 3.5% and 4.6%, respectively. We have also found that the risk of liver fibrosis by APRI score is significantly increased among HBV coinfected HIV patients.

The reported prevalence of HBV/HIV coinfection in this study is comparable to other previous reports with similar settings from Tanzania (6.2%), Kenya (6.0%), and Uganda (6.7%) [3, 11, 12]. It is slightly lower than 7.8% which is the median prevalence rate reported in a SSA multianalysis survey [2]. In this study East African countries however were found to have the lowest HBV coinfection with median rate of 4.1% as compared to South and West African countries. Moreover the established baseline characteristics of the risky population to HBV coinfection in the index study are similar to the findings from previous reports: young age [5], advanced HIV disease (stage 3 or 4) [13], low CD4 count [14], and elevated AST and ALT levels [15]. Due to shared transmission mechanisms with their synergetic clinical effects, guidelines for management of HIV and AIDS recommend screening for HBV in all HIV-infected patients [16]. Nevertheless, a particular attention should be paid to this subgroup of patients including a close follow-up and further investigation since they are additionally at high risk of having occult hepatitis B (OHB) infection which refers to the presence of plasma HBV DNA without HBV surface antigen (HBsAg) but with core antibodies (anti-HBc) [17, 18].

Liver fibrosis detected by noninvasive tests (NIT) among HIV-infected patients in SSA has been rarely reported. To the best of our knowledge, there is only one published study from Tanzania which showed an overall prevalence rate of 9.1% and a cirrhosis rate of 5.3% by APRI score. In this study a highest rate of fibrosis was observed among HIV/HBV coinfected patients as compared to their HIV monoinfected counterparts (14.2% versus 8.7%, *P* = 0.03). Being clinically important, it was further found that the score regressed at 24 months following use of ART [9] though this was not assessed in the current study. Similar findings have also been reported in a number of other studies [19–21]. In another study from Nigeria, for instance, liver fibrosis was also more common among HBV coinfected HIV patients. In this study the reported liver fibrosis was higher by both APRI score (17% versus. 4%, *P* = 0.02) and FIB-4 score (13% versus. 2%, *P* = 0.001) among HBV coinfected HIV patients compared to HIV monoinfected participants [22]. A similar trend was observed in the index study where we found that HIV/HBV coinfected participants had eight times higher risk of liver fibrosis than the HBV monoinfected group. However, the justification for a discrepancy of prevalence from a previous study in Tanzania [9] could not be ascertained.

Liver biopsy remains the gold standard for diagnosing and staging histological changes despite its shortcomings such as invasiveness, bleeding, sampling error, and high-expertise need with infrastructures [23, 24]. In this regard, other noninvasive methods were explored including APRI and FIB-4 as most commonly studied alternative tools even though their diagnostic accuracy for liver fibrosis among HIV-infected patients is still inconsistent [25–28]. Though one previous study comparing the performances of these tools indicated superior accuracy of APRI over FIB-4 score [29], this was not a case in our study as they both showed an excellent agreement (Kappa of 0.967). Despite these variations, several international guidelines still recommend the utilization of these tools to reduce the need of liver biopsy [30, 31]. In its recent recommendations for the diagnosis and management of liver disease, WHO strongly encourages the use of these tools in countries where liver biopsy is not readily feasible in the decision of treatment as the benefit of use of these tools outweighs most potential harms of biopsy methods [7].

APRI score might be overestimated in HIV-infected patients due to conditions like HIV-induced thrombocytopenia or hepatitis due to other drugs like antituberculosis and local herbs that might elevate AST. This scenario was unlikely to have occurred in our cohort as majority (92%) of the patients had normal platelets with high median value of 247,000 (normal: 150,000) cells/*μ*l with AST and ALT values which were within normal ranges.

Assessment of other potential confounders like alcohol intake, medication history including local herbs, and other forms of hepatitis on liver fibrosis was beyond the scope of this study. Also, the clinical outcome of liver fibrosis on receipt of ART could not be determined since patients were not followed up. Moreover the lack of data on other parameters of hepatitis infection like HBV DNA, HBV envelope antigen, and HBV core antibody levels precludes further comments on associations with significant fibrosis/cirrhosis in coinfected patients.

## 5. Conclusion

The current study has conclusively demonstrated that a substantial number of ART naïve HIV patients present with liver fibrosis and majority are coinfected with HBV infection. We have also revealed that younger people with advanced HIV disease and elevated liver transaminases were more likely to present with HBV coinfection. These findings however should be interpreted with caution due to insufficient number of comparison patients in HIV/HBV coinfected group that might have affected the statistical associations. Despite this major shortcoming, we believe that our findings which are rare in the country are important and relevant as they provide a broad picture on the situation locally and baseline information for further studies on the subject. These findings promote utilization of NIT in routine clinical care in our setting which can potentially detect the liver fibrosis at its earliest stage and manage it timely. There is also a need to prepare a national guideline for screening and management of liver diseases based on the locally available resources. Furthermore, longitudinal study is recommended to ascertain the outcome of these patients with liver fibrosis following the use of ART.

## Figures and Tables

**Table 1:** Baseline sociodemographic and clinical characteristics of 743 enrolled ART naïve patients.

Characteristic	All patients (*n* = 743) Number (%) or median (IQR)	HIV-monoinfected (*n* = 694) Number (%) or median (IQR)	HBV/HIV coinfected (*n* = 49) Number (%) or median (IQR)	Odds ratio (95% CI)	*P* value
Sex					
Males	259 (34.9)	242 (34.8)	17 (34.7)	0.99 (0.54–1.82)	0.98
Age (years)	37 (IQR 30–44)	37 (30–44)	38 (30–43)	0.99 (0.97–1.02)	0.83
≥39	329 (44.28)	309 (44.52)	20 (40.82)	1.26 (1.01–1.45)	**0.002**
Age group ≥ 39	45 (41–51)	45 (41–51)	47.5 (41–51)	1.00 (0.95–1.06)	0.91
Age group < 39	31 (25–35)	31 (25–35)	30 (26–36)	1.01 (0.95–1.1)	0.75
Marital status					
Single	189 (25.4)	175 (25.2)	14 (28.6)	1.19 (0.62–2.26)	0.61
Married	300 (40.4)	281 (40.5)	19 (38.8)	0.93 (0.51–1.69)	0.81
Divorced	132 (17.8)	121 (17.4)	11 (22.5)	0.37 (0.68–2.78)	0.38
Widowed	122 (16.4)	117 (16.9)	5 (10.2)	0.56 (0.22–1.44)	0.23
AIDS stage					
1 or 2	386 (51.9)	367 (52.9)	19 (38.8)		
3 or 4	357 (48.1)	327 (47.1)	30 (61.2)	1.77 (0.98–3.21)	**0.006**
Baseline CD4 (cells/*µ*l)	256 (123–458)	266 (132–465)	158 (88.5–295)	1.46 (0.76–2.84)	**0.02**
CD4 < 200	251 (33.8)	218 (31.4)	33 (67.4)	4.50 (2.43–8.36)	**<0.001**
CD4 ≥ 200 group	400 (290–591)	513 (308–583)	399 (286–594)	1.00 (0.99–1.00)	1.00
CD4 < 200 group	91 (37–151)	123 (49–162)	87 (36–150)	1.00 (0.99–1.01)	0.23
Liver enzymes (IU/l)					
AST	26 (19.5–37)	26 (19.6–37)	30.2 (18.8–57.3)	1.01 (1.00–1.02)	**<0.001**
AST > 40	254 (34.2)	230 (33.1)	24 (48.5)	1.94 (1.08–3.46)	**0.03**
ALT	19 (13–30)	18.3 (13–29)	25.9 (13–45)	1.01 (1.00–1.02)	**0.02**
ALT > 41	203 (27.32)	182 (26.22)	21 (42.86)	2.11 (1.17–3.81)	**0.01**
Platelets (cells/ul)	247 (189–330) × 10^3^	250 (191–331) × 10^3^	203 (170–275) × 10^3^	1.04 (0.83–1.05)	0.05
<150 × 10^3^	63 (8.5)	56 (8.1)	7 (14.3)	0.53 (0.23–1.23)	0.14

**Table 2:** APRI and FIB-4 scores results among 456 patients with available results for analysis.

		APRI	
		HIV monoinfected (*n* = 440)	HIV/HBV coinfected (*n* = 16)
		≤1.5	>1.5
FIB-4	>3.25	10 (2.3%)	11 (68.8%)
	≤3.25	430 (97.7%)	5 (31.3%)

Kappa = 0.967.

**Table 3:** Prevalence of liver fibrosis by APRI and FIB-4 scores among HIV monoinfected and HIV/HBV coinfected patients.

		All patients (*n* = 456)	HIV monoinfected (*n* = 427)	HBV/HIV (*n* = 29)	OR (95% CI)	*P* value
APRI score		0.29 (0.19–0.44)	0.28 (0.19–0.43)	0.45 (0.23–0.85)	2.21 (1.44–3.38)	<0.001
	APRI > 1.5	16 (3.51)	11 (2.58)	5 (17.24)	7.88 (2.53–24.49)	<0.001
	APRI > 2.0	10 (2.19)	6 (1.41)	4 (13.79)	22.1 (4.7–104.1)	<0.001
FIB-4 score		0.97 (0.65–1.54)	0.96 (0.65–1.50)	1.34 (0.82–2.09)	1.24 (1.01–1.51)	0.04
	FIB-4 > 3.25	21 (4.61)	19 (4.49)	2 (6.90)	1.59 (0.35–7.18)	0.55
